# The central role of DNA damage and repair in CAG repeat diseases

**DOI:** 10.1242/dmm.031930

**Published:** 2018-01-01

**Authors:** Thomas H. Massey, Lesley Jones

**Affiliations:** Institute of Psychological Medicine and Clinical Neurosciences, MRC Centre for Neuropsychiatric Genetics and Genomics, Hadyn Ellis Building, Cardiff University, Cardiff, CF24 4HQ, UK

**Keywords:** CAG repeat, DNA damage, DNA repair, Huntington's disease, Spinocerebellar ataxia

## Abstract

Diseases such as Huntington's disease and certain spinocerebellar ataxias are caused by the expansion of genomic cytosine-adenine-guanine (CAG) trinucleotide repeats beyond a specific threshold. These diseases are all characterised by neurological symptoms and central neurodegeneration, but our understanding of how expanded repeats drive neuronal loss is incomplete. Recent human genetic evidence implicates DNA repair pathways, especially mismatch repair, in modifying the onset and progression of CAG repeat diseases. Repair pathways might operate directly on repeat sequences by licensing or inhibiting repeat expansion in neurons. Alternatively, or in addition, because many of the genes containing pathogenic CAG repeats encode proteins that themselves have roles in the DNA damage response, it is possible that repeat expansions impair specific DNA repair pathways. DNA damage could then accrue in neurons, leading to further expansion at repeat loci, thus setting up a vicious cycle of pathology. In this review, we consider DNA damage and repair pathways in postmitotic neurons in the context of disease-causing CAG repeats. Investigating and understanding these pathways, which are clearly relevant in promoting and ameliorating disease in humans, is a research priority, as they are known to modify disease and therefore constitute prevalidated drug targets.

## Abstract (precis)

**Summary:** Recent genetic discoveries have suggested that DNA repair processes are central to CAG repeat disease pathogenesis. In this review, we consider how DNA damage and repair could trigger, and also result from, CAG repeat expansion to drive neurodegeneration.

## Introduction

Expanded cytosine-adenine-guanine (CAG) trinucleotide repeats in the exons of certain genes can induce neurodegeneration in the central nervous system (CNS). Diseases caused by expanded CAG repeats include Huntington's disease (HD), which has a prevalence of approximately 1 in 8000 in populations of European descent (Evans et al., 2013; Fisher and Hayden, 2014), and various spinocerebellar ataxias (SCAs), which are individually very rare but have a combined prevalence of ∼1 in 40,000 in European/Asian populations (Ruano et al., 2014). These dominantly inherited diseases are all characterised by slow, progressive neuronal loss over 10-20 years, leading to worsening disability and, eventually, death. Specific clinical manifestations depend on the genes and cell types affected by the repeat expansion but, despite the phenotypic variation between these diseases, a common underlying molecular pathology seems likely. In support of this hypothesis, recent human genetic data suggest that DNA repair pathways are central to the pathogenesis of CAG repeat diseases (see Glossary, Box 1) (Bettencourt et al., 2016; GeM-HD Consortium, 2015). In this review, we consider how DNA damage and repair pathways could potentially mediate CAG repeat-driven pathology in CNS neurons. A better understanding of these cellular mechanisms could identify novel drug targets, an urgent requirement in the field given that there are currently no disease-modifying treatments for any CAG repeat disorder.

**Box 1. Glossary**

**Autophagy:** mechanism by which cells recycle macromolecules through lysosomes.

**Base excision repair (BER):** pathway that senses and repairs small, nondistorting base lesions in DNA (e.g. arising from oxidative damage). Two major subpathways are known: short-patch (SP-BER) and long-patch (LP-BER) relating to the amount of gap-filling DNA repair synthesis required (Fig. 2).

**CAG repeat disorder:** disease caused by a number of repeated, consecutive CAG trinucleotide units in DNA over a threshold length.

**Cockayne syndrome B protein (CSB):** ATPase with multiple functions in DNA repair as well as roles in chromatin remodelling, transcription and mitochondrial function. Mutations in CSB cause ∼75% of Cockayne syndrome, a disease of neurodevelopmental abnormalities, neurodegeneration and premature ageing.

**DNA damage response (DDR):** network of overlapping pathways in cells involved with DNA damage signalling and repair, integrated with the cell cycle. Encoded by >450 genes in humans.

**Fig. 1.**
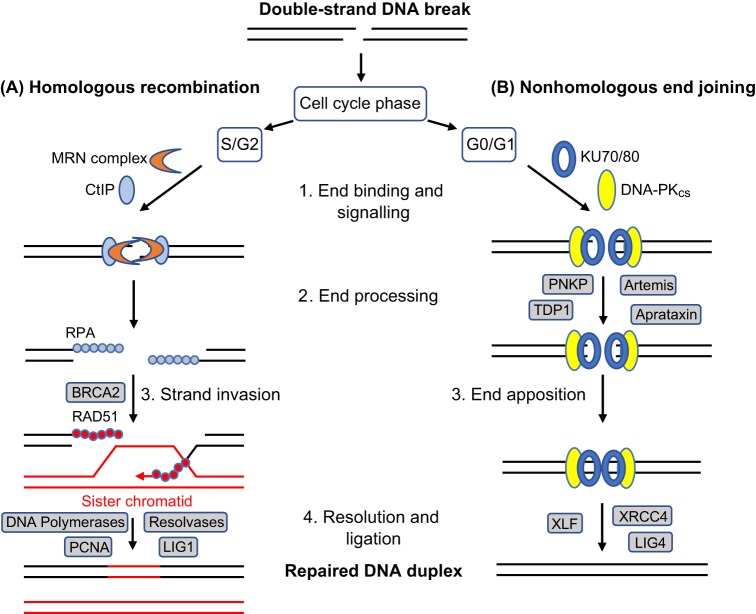
**DNA double-strand break repair pathways in neurons, highlighting key similarities and differences.** (A) Homologous recombination is utilised in S or G2 phase of dividing neuronal progenitors. DNA ends are processed by MRN complexes and other proteins to produce 3′-overhangs coated by RPA proteins. BRCA2 catalyses the exchange of RPA for RAD51, thus enabling invasion of the sister chromatid and error-free repair. (B) Nonhomologous end joining is utilised by postmitotic neurons in G0 or G1. DNA ends are bound by KU70/80, leading to the recruitment of DNA-PK_cs_. End processing is carried out by various enzymes including PNKP and Artemis (DCLRE1C), and then ends are ligated by LIG4-XRCC4. This form of repair preserves genomic integrity but can be error prone. Proteins at key commitment points are shown in colour, others in grey boxes. Some factors involved in double-strand break repair and cell-cycle regulation are omitted for clarity. BRCA2, breast cancer type 2 susceptibility protein; CtIP, C-terminal binding protein 1 interacting protein; DNA-PK_cs_, DNA-dependent protein kinase, catalytic subunit; MRN complex, MRE11-RAD50-NBS1 complex; LIG, DNA ligase; PCNA, proliferating cell nuclear antigen; PNKP, polynucleotide kinase 3′-phosphatase; RPA, replication protein A; TDP1, tyrosyl-DNA phosphodiesterase 1; XLF, XRCC4-like factor; XRCC4, X-ray repair cross-complementing 4.

**Double-strand break repair (DSBR):** mechanism for ensuring genomic integrity following double-strand DNA breakage. Homologous recombination and nonhomologous end joining are the two main pathways (Fig. 1).

**Genetic modifier:** genetic variant that is not directly causative for a disease, but can affect the phenotype when occurring together with the disease-causing mutation.

**Genome-wide association study (GWAS):** unbiased, observational, pan-genome screen for common genetic variants associated with a particular disease or trait.

**Lynch syndrome:** a cancer predisposition syndrome caused by mutations in mismatch repair genes such as *MSH2* and *MLH1*. Mutation carriers are at increased risk of colorectal and other cancers.

**Medium spiny neurons (MSNs):** inhibitory GABA-ergic interneurons that make up >95% of striatal neurons in the human brain. First neurons to degenerate in Huntington's disease.

**Fig. 2.**
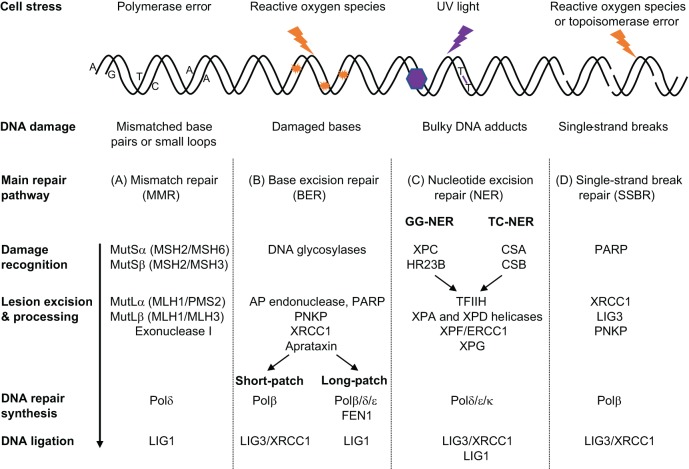
**Similarities and differences between the principal mammalian single-strand DNA damage repair pathways.** Examples of cell stressors are shown, with resultant DNA damage. DNA repair proceeds through a conserved general mechanism of damage recognition, lesion excision and processing, DNA repair synthesis and ligation of DNA ends, as shown from top to bottom. Components vary between pathways although there is considerable overlap. The four main repair pathways for single-strand DNA lesions are shown, with the key proteins involved. (A) Mismatch repair (MMR). (B) Base excision repair (BER). (C) Nucleotide excision repair (NER). (D) Single-strand break repair (SSBR). AP endonuclease, apurinic/apyrimidinic endonuclease 1; CSA/CSB, Cockayne syndrome protein A/B; ERCC1, excision-repair cross-complementing 1; FEN1, flap endonuclease 1; GG-NER, global genomic nucleotide excision repair; HR23B, human RAD23 homologue B; LIG, DNA ligase; MLH, MutL protein homologue; MSH, MutS protein homologue; PARP, poly(ADP-ribose) polymerase; PMS2, postmeiotic segregation increased 2; PNKP, polynucleotide kinase 3′-phosphatase; Pol, DNA polymerase; TC-NER, transcription-coupled nucleotide excision repair; TFIIH, transcription factor IIH; UV, ultraviolet; XPA/XPC/XPD/XPF/XPG, DNA repair proteins in different xeroderma pigmentosum (XP) complementation groups; XRCC1, X-ray repair cross-complementing 1.

**Mismatch repair (MMR):** pathway that is canonically involved in strand-specific repair of mismatched base-pairs arising from DNA replication errors in proliferating cells. Recent data indicate broader repair functions, including in nondividing cells such as neurons (Fig. 2).

**MutL complex:** protein dimer involved in mismatch processing downstream of MutS complex in the MMR pathway (Fig. 2).

**MutS complex:** protein dimer responsible for initial mismatch recognition in MMR. Two complexes with overlapping substrate specificities exist in eukaryotes: MutSα (MSH2/MSH6) and MutSβ (MSH2/MSH3), the latter found predominantly in neurons (Fig. 2).

**Nucleotide excision repair (NER):** a DNA repair pathway that senses and repairs bulky lesions (e.g. photoproducts from UV irradiation) by removing and replacing damaged nucleotides. Two major subpathways are known: global genomic (GG-NER), involved in pan-genomic DNA repair, and transcription-coupled (TC-NER), involved in repair in actively transcribed genes. These subpathways differ in damage sensing, but share downstream repair processes (Fig. 2).

**Poly(ADP-ribose) polymerases (PARP):** family of nuclear enzymes that detect single-strand breaks in DNA and signal to downstream repair factors through ADP ribosylation of target proteins.

**Reactive oxygen species (ROS):** byproducts of oxidative cellular metabolism that can react with, and damage, DNA and other macromolecules. Examples include superoxide radicals and hydrogen peroxide.

**Repeat-associated non-ATG (RAN) translation:** noncanonical mRNA translation initiated by tandem repeats rather than the ATG codon and leading to toxic homopolymeric proteins in cells.

**Single-strand break repair (SSBR):** a pathway that senses and repairs breaks in one strand of the DNA double helix. Shares components with base excision repair (Fig. 2).

**SPO11:** tyrosine recombinase that initiates recombination in meiosis I by inducing programmed double-strand breaks in DNA.

**Synthetic lethality:** interaction between two genes where deficiency of either alone is tolerated, but simultaneous deficiency of both is lethal to the cell. Harnessed in screens for novel (cancer) therapeutics.

**Topoisomerase:** enzyme that regulates the supercoiling of DNA by cleavage and re-ligation reactions on one strand (Type I) or both strands (Type II) of the double helix.

**V(D)J recombination:** a mechanism of programmed DNA strand breakage and repair that occurs in maturing B cells and T cells to generate antibody and T cell receptor diversity, respectively.

## DNA damage and repair in the CNS

DNA is continually damaged and repaired in all living cells. Intricate DNA repair mechanisms have evolved in parallel with increasing genome complexity in order to preserve genetic information (O'Brien, 2006). However, inaccurate repair can be mutagenic, while failed repair can threaten the integrity of the genome. Different cells sustain different types of DNA damage, and various overlapping mechanisms within the overarching DNA damage response (DDR; see Glossary, Box 1) are required for effective repairs (Jackson and Bartek, 2009; Pearl et al., 2015). The adult postmitotic neurons that degenerate in CAG repeat diseases sustain and repair particular types of DNA damage, as we discuss below.

### DNA damage

It has been estimated that each mammalian cell sustains as many as 10,000 single-strand and 10-50 double-strand DNA breaks per day (Madabhushi et al., 2014). Exogenous sources of DNA damage, such as UV light, ionising radiation and chemical mutagens, predominantly affect exposed and dividing cells, but in neurons, and especially in neurons of the CNS, endogenous metabolic processes are more relevant sources of DNA damage. The high oxygen demands of the brain (20% of total body oxygen consumption, but only 2% of body mass) expose its cells to numerous reactive oxygen species (ROS; see Glossary, Box 1) produced by normal mitochondrial respiration (Cooke et al., 2003), and further oxidative damage can arise as a result of inflammation. Over 100 different types of DNA base damage have been identified as being caused by ROS, the most abundant of which is 8-oxo-2′-deoxyguanosine (8-oxo-dG). One study of human lymphocytes found 8-oxo-dG at a steady state of ∼10,000 damaged bases per cell nucleus (Ohno et al., 2006). This altered base can be premutagenic in replicating cells and has inhibitory effects on transcription in postmitotic neurons (Iyama and Wilson, 2013). In addition to ROS, cellular metabolism generates endogenous alkylating compounds (e.g. S-adenosyl methionine), lipid peroxidation products, and reactive nitrogen and carbonyl species that can directly damage DNA. DNA is also susceptible to hydrolysis, which can directly cause base loss or deamination, particularly in single-stranded regions. CAG repeats might be especially susceptible to damage as hydrolytic depurination (i.e. loss of A or G) and cytosine deamination are frequent events (Lindahl, 1993).

In addition to pathological DNA damage, there is increasing recognition of the role of physiological, ‘programmed’ DNA strand breakage. For example, outside the CNS, the generation of antibody and T cell receptor diversity depend on programmed double-strand breaks, and their subsequent repair, as part of V(D)J recombination (see Glossary, Box 1) (Slean et al., 2008), and meiotic crossing over is initiated by SPO11-induced double-strand breaks (see Glossary, Box 1) (Keeney et al., 2014). Topoisomerases, which can introduce temporary single- or double-strand breaks in DNA to regulate supercoiling (see Glossary, Box 1), are essential for the expression of long genes and are particularly important in the brain, where expressed genes are longer than elsewhere in the body (Gabel et al., 2015; King et al., 2013; Zylka et al., 2015). Recently, it has also been shown that neuronal activity in cells and in animals can trigger topoisomerase-induced double-strand DNA breaks in the promoters of neuronal early-response genes (Ju et al., 2006; Madabhushi et al., 2015; Suberbielle et al., 2013). These DNA breaks might relax topological constraints and stimulate promoter activity. Topoisomerase mutations can lead to various neurodevelopmental and neurodegenerative conditions, highlighting the importance of torsional regulation of DNA in brain function (King et al., 2013; McKinnon, 2016).

Therefore, neuronal DNA strands are continually broken and repaired *in vivo*. The tight regulation of DNA repair is crucial for maintaining neuronal gene expression and function; indeed, many Mendelian neurological diseases result directly from defects in the DNA damage response (Madabhushi et al., 2014).

### DNA repair

Unrepaired DNA damage can have profound consequences on cells. For example, point mutations or chromosomal rearrangements can lead to cancer in dividing cells, such as glia, and can induce cell death in nondividing cells, such as CNS neurons. Moreover, lesions or strand breaks can stall DNA and RNA polymerases, leading to impaired replication or transcription, respectively, and potentially triggering cell death or senescence (Hoeijmakers, 2009). Therefore, an elaborate damage response has evolved to identify and repair DNA damage in conjunction with cell cycle regulation. The DDR involves >450 genes in humans, with subsets of these genes deployed depending on the type of DNA damage, type of cell, stage of cell cycle and stage of organism development (Pearl et al., 2015).

Human CNS development involves the proliferation and subsequent migration and differentiation of neural progenitor cells, beginning a few weeks after conception and continuing to ∼6 months after birth (Rulten and Caldecott, 2013). Most neurons then enter a postmitotic phase (and are required to survive for a lifetime), although there is clearly a high turnover of molecules within these cells. A small subset of neurons in the human hippocampus and lateral ventricle of the mature CNS can divide and contribute to ongoing neurogenesis (McKinnon, 2013). The phase of cell cycle determines which DNA repair pathways are utilised in neurons. For example, dividing progenitors in S/G2 phase use accurate homologous recombination (HR) for double-strand break repair (see Glossary, Box 1) and replication fork maintenance (Fig. 1). Mutations in these repair systems are embryonic lethal or lead to profound neurodevelopmental defects (McKinnon, 2013; Rulten and Caldecott, 2013). By contrast, mature postmitotic neurons in G0/G1 rely upon less accurate nonhomologous end joining (NHEJ) for double-strand break repair in the absence of a sister chromatid (Fig. 1). In addition, DNA damage in these neurons is predominantly single stranded, which can lead to the inhibition of transcription, as well as compromised genomic integrity. Pathways such as single-strand break repair (SSBR; see Glossary, Box 1) and transcription-coupled nucleotide excision repair (TC-NER; see Glossary, Box 1) are essential to cellular function, and mutations in these pathways lead to late neurodevelopmental defects, such as microcephaly, and, more commonly, neurodegeneration (McKinnon, 2013; Rulten and Caldecott, 2013). The type and location of affected neurons determine the clinical phenotypes observed (Madabhushi et al., 2014).

Many parts of the DDR are highly conserved from prokaryotes to eukaryotes, with extra layers of regulation and redundancy found in higher organisms. Individual linear repair pathways, such as base excision repair (BER; see Glossary, Box 1), nucleotide excision repair (NER; see Glossary, Box 1) and mismatch repair (MMR; see Glossary, Box 1), involve a series of analogous steps: lesion recognition, repair factor recruitment, lesion excision leading to DNA strand breakage, processing of DNA ends and DNA synthesis, to complete repair (Fig. 2). In addition, it is increasingly recognised that there is significant redundancy between pathways, presumably arising through divergent evolution in order to repair a wide range of lesions whilst maintaining genomic integrity (O'Brien, 2006; Pearl et al., 2015). Pathways can also be involved in noncanonical repairs. For example, MMR enzymes, which usually act at replication forks in dividing cells to correct DNA polymerase errors, have been shown to recognise and repair mispaired bases in nondividing yeast cells (Rodriguez et al., 2012). Therefore, DNA repair systems have activities that depend not only on the type of DNA damage but also on the cell/tissue context, the background genetics of the cell, and the prevailing environmental conditions. CAG repeats in the DNA of adult postmitotic neurons constitute a particular substrate for repair systems, and the regulation of these repair activities is entwined with CAG repeat disease pathogenesis, as discussed below.

## Repetitive DNA and CAG repeat disorders

Recent estimates suggest that >65% of the human genome consists of repetitive elements, ranging from microsatellites (2-6 base pair tandem repeats) up to whole genes (e.g. rDNA gene arrays) (Hall et al., 2017; de Koning et al., 2011). These elements can be coding or noncoding and have a range of essential functions in cells, including at centromeres and telomeres. Microsatellites are known to be common and hypermutable in both prokaryotic and eukaryotic genomes (Bichara et al., 2006). Their mutability can aid adaptation to changing environments, particularly in microorganisms, and might have a role in the regulation of gene expression in eukaryotes (Bichara et al., 2006; Gymrek et al., 2015). The processive mechanisms of DNA and RNA polymerases on unwound DNA mean that tandem repeats can readily adopt noncanonical conformations in DNA, such as slipped strands, hairpin loops, G-quadruplexes and R-loops (Mirkin, 2007; Neil et al., 2017). These structural perturbations of DNA have been implicated in both the normal regulation of cellular functions, such as chromatin organisation and gene expression, and in the aberrant DNA processing that can lead to genomic instability. Repetitive genomic loci are often polymorphic but cells have homeostatic mechanisms to maintain fairly stable repeat lengths in DNA based on structural stability, protein binding and reaction kinetics (Hall et al., 2017; Lee and McMurray, 2014). However, sometimes these mechanisms fail and repeats expand or contract significantly, often with resulting pathology.

Trinucleotide repeat disorders are human diseases that are defined by expanded tandem arrays of three-nucleotide units in the transcribed regions of a diverse range of genes (Budworth and McMurray, 2013). Strikingly, all of these diseases have at least some element of neurological dysfunction suggestive of a specific need to regulate trinucleotide repeats tightly in the nervous system (Orr and Zoghbi, 2007). Diseases caused by expanded tandem CAG repeats in exons constitute a subset of the broader trinucleotide repeat disorder group, and are linked by central neurodegeneration, as well as DNA sequence. They are considered in more detail below.

The first disease shown to be caused by an expanded CAG repeat was spinal and bulbar muscular atrophy (SBMA) in 1991 (Spada et al., 1991). Shortly afterwards, various other dominantly inherited neurodegenerative conditions were also linked to expanded CAG tracts, including HD, dentatorubral-pallidoluysian atrophy (DRPLA), and some spinocerebellar ataxias (SCA1,2,3,6,7,12,17) (Koide et al., 1994; Nagafuchi et al., 1994; Orr et al., 1993; The Huntington's Disease Collaborative Research Group, 1993). Although these diseases all share a common underlying mutation type, genotype-phenotype relationships are not straightforward. Expanded CAG repeats can variably cause autosomal dominant ataxia (SCAs), chorea (HD or DRPLA), or neither (SBMA), in association with a variety of other symptoms. Only a small subset of SCAs are caused by CAG repeats; of the 43 autosomal dominant SCAs described to date, just seven have been attributed to CAG repeats (Synofzik and Schüle, 2017). In addition, there is considerable phenotypic diversity between even those SCAs caused by CAG repeat expansion: for example, SCA6 presents as a fairly ‘pure’ ataxia, whereas SCA7 often has associated retinal degeneration (Sun et al., 2016; Synofzik and Schüle, 2017). However, even though spinocerebellar degeneration can be caused by a wide variety of CAG repeat and non-CAG mutations in disparate genes, there might be a shared molecular pathogenesis. In support of this hypothesis, many SCA gene products interact with, and presumably mediate their effects through, a limited set of intracellular proteins, the ‘ataxia interactome’ (Kahle et al., 2011; Lim et al., 2006). Pathogenic CAG expansions are also found in some patients with autosomal dominant chorea as part of their clinical presentation; for example, in HD (with psychiatric, behavioural and cognitive symptoms) (Bates et al., 2015) or in DRPLA (with myoclonic epilepsy and ataxia) (Wardle et al., 2009). Again, partially shared clinical phenotypes and underlying mutations hint at a common pathology, and there might be a broader commonality with the CAG repeat SCAs, as exemplified by SCA17, which is also known as Huntington's disease-like 4 (HDL-4), owing to its clinical presentation (Gövert and Schneider, 2013).

Pathogenic CAG repeat expansions are found in the exons of different genes for each of the different diseases (Table 1). Wild-type repeat numbers range from 4 to ∼40 and are polymorphic at each locus. When transcribed and translated, a tandem CAG repeat tract, (CAG)_*n*_, encodes a polyglutamine stretch in protein, and this polypeptide could have important effects in cells outwith the function of the endogenous protein in which it sits (Ashkenazi et al., 2017; Fujita et al., 2013). Expansion of the tandem CAG repeat over a threshold is necessary and sufficient for all the CAG repeat diseases, suggesting a toxic gain of function. The toxic threshold is usually of the order of 35-45 tandem repeats, although with some variation; for example, the disease threshold is shorter in SCA6 (>19 repeats) and longer in SCA3 (>60 repeats) (Table 1) (Durr, 2010). Once over the disease threshold, longer repeat lengths are associated with earlier symptom onset, although there is considerable variation (Bates et al., 2015; Durr, 2010). Toxicity is conferred by the expanded repeat, but the mechanisms by which CAG repeat expansion leads to specific neurodegeneration remain incompletely understood. Theoretically, CAG-containing DNA and/or mRNA and/or polyglutamine-containing proteins could be pathogenic. Repetitive DNA elements affect gene expression (Gymrek et al., 2015), and there is also evidence of the toxicity of both CAG-containing mRNA and polyglutamine in cells (Cheng et al., 2015; Rué et al., 2016). Additionally, recent evidence from human HD brains has suggested that sense and antisense mRNAs from (CAG)_*n*_ can be translated in all possible frames by noncanonical repeat-associated non-ATG (RAN) translation to produce toxic homopolymeric proteins (see Glossary, Box 1) (Bañez-Coronel et al., 2015). Although all underpinned by similar CAG repeat expansions, the different disease phenotypes are associated with the selective degeneration of different brain cell types; for example, cerebellar Purkinje cells are affected in SCAs and striatal medium spiny neurons (MSNs; see Glossary, Box 1) in HD. The reasons for this selectivity are unclear, but emphasise how gene expression, protein context and cell type can all influence CAG repeat pathology.

**Table 1.**
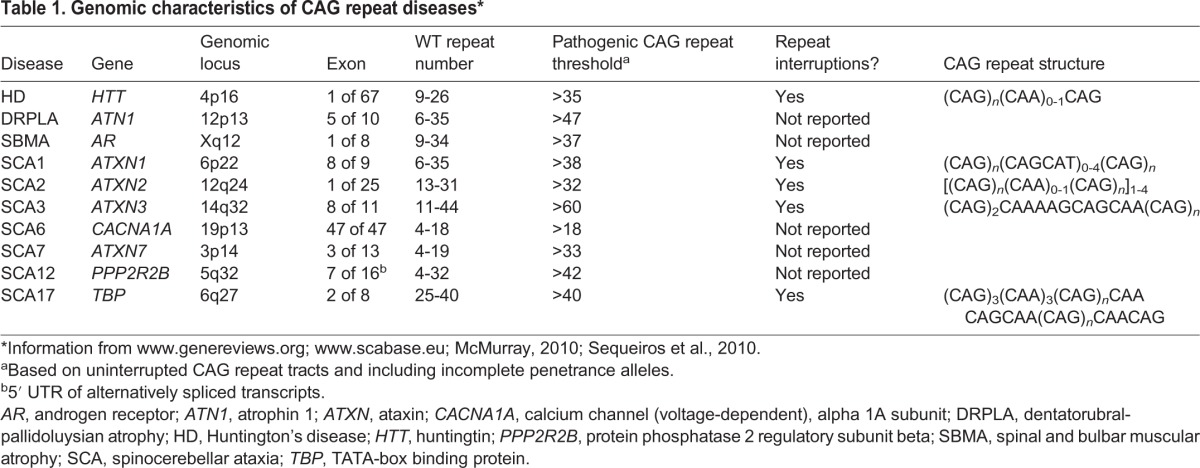
Genomic characteristics of CAG repeat diseases*

Given their shared causative repeat expansions and overlapping clinical phenotypes, the CAG repeat disorders might be linked by a common pathogenesis at the DNA level, involving DNA damage and repair in neurons. We discuss the intersection of CAG repeat disorders with DNA repair in more detail below.

## CAG repeat disorders and DNA repair

Links between DNA repair defects and neurodegenerative diseases have been known for many years. Fibroblasts and lymphocytes cultured from patients with HD, Alzheimer's disease, Parkinson's disease and amyotrophic lateral sclerosis have all been shown to be sensitive to DNA damage induced by ionising radiation or exogenous chemical mutagens (Moshell et al., 1980; Robison and Bradley, 1984; Scudiero et al., 1981). It has been suggested that accumulation of DNA damage as a result of inadequate DNA repair could cause neurodegeneration, although it has been difficult to discriminate between this hypothesis and the accrual of DNA damage caused by other pathological cellular dysfunction (Robison and Bradley, 1984). The discovery of neurodegenerative CAG repeat disorders, and the apparent similarity of their repeat length variation to that observed in microsatellites of some colorectal cancers, led to a second line of investigation: the role of DNA repair in the modulation of CAG repeat length. However, microsatellite instability is observed throughout the genome in the MMR-deficient tumours of Lynch syndrome, a cancer-predisposition disorder (see Glossary, Box 1), alongside a globally elevated mutation rate. By contrast, HD/SCA patients only seem to show repeat number variation at a disease-specific, expanded CAG repeat locus (Goellner et al., 1997; Slean et al., 2008). These patients also have a significantly reduced incidence of cancer [e.g. a standardised incidence ratio of 0.47 in the largest study of HD (Ji et al., 2012)].

**Fig. 3.**
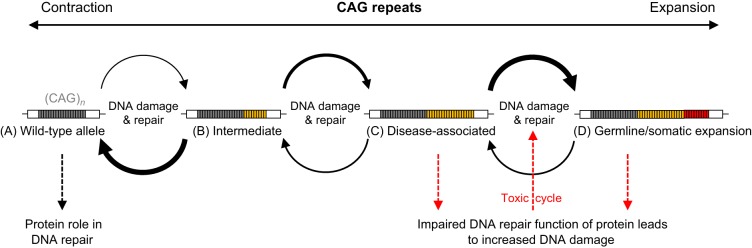
**DNA damage and repair can affect CAG repeat length with downstream effects on disease pathogenesis.** CAG repeats in DNA are unstable, and cycles of DNA damage and repair can lead to changes in repeat length. (A) Wild-type length repeats can expand to (B) intermediate lengths, stochastically. These will mostly be repaired to wild-type length (black, bold arrow from B to A), perhaps through a dedicated pathway, but a small number will expand further (C) into the disease-associated range in gametes. (D) Once over the disease-causing threshold, repeats are predisposed to expand further (black, bold arrow from C to D) in both germline and somatic cells. In addition to the role of DNA repair in repeat length changes, some genes containing CAG repeats encode proteins with roles in DNA repair. Expanded repeats can impair the functions of these DNA repair proteins, leading to the accrual of DNA damage in neurons and to a toxic cycle of DNA damage/repair and repeat expansion (red dashed arrows). DNA repair variants associated with earlier or later disease onset could affect any of these processes.

Once the disease-causing threshold is crossed, CAG repeat length has an inverse relationship with the age at symptom onset. However, in HD, the most well-studied CAG repeat disorder, repeat length only explains ∼50% of the observed variation in age at symptom onset. Studies of the large Venezuelan HD kindreds indicated that as much as 40% of the remaining variation was heritable, suggesting that background genetic variants can have a large influence on when symptoms start (Wexler et al., 2004). In order to identify these genetic modifiers (see Glossary, Box 1), a genome-wide association study (GWAS; see Glossary, Box 1) was recently performed using data from just over 4000 HD patients, to look for loci associated with earlier or later onset HD than predicted by CAG repeat length alone (GeM-HD Consortium, 2015). This study identified variants at a number of loci with significant associations with the age at symptom onset. Many of these variants are in, or near, the genes that encode components of DDR pathways, and particularly those involved with MMR (GeM-HD Consortium, 2015). Subsequent work showed that many of the same genetic modifiers are significant in other CAG repeat disorders, suggesting that there is a common pathogenic mechanism driving disease onset, perhaps at the level of the somatic CAG repeat (Bettencourt et al., 2016). Furthermore, a comparison of disease progression with genotype in a sample of HD patients that had been phenotyped in detail showed a genome-wide significant signal in *MSH3*, a MMR gene (Hensman-Moss et al., 2017). Collectively, these results were the first to link human CAG repeat disorder phenotypes directly to DNA repair, and corroborated many earlier results from model systems. The simplest explanation for the genetic data is that DNA repair variants directly affect repeat number in individuals, but it is also possible that expanded (CAG)_*n*_ could exacerbate DNA repair defects (Fig. 3). In the sections below, we explore the evidence that links CAG repeat diseases and DNA repair either at the level of the CAG repeat in the genome, or as a downstream consequence of an expanded CAG repeat.

## CAG repeats in the genome

Expansion of a tandem CAG repeat in genomic DNA over a threshold number is absolutely required for each of the CAG repeat disorders (Table 1). Long repeats are intrinsically unstable, as shown in cell-free and unicellular systems, and their dynamics in neurons and gametes are linked to disease pathology.

### Intrinsic instability of CAG repeat number

Biophysical studies *in vitro* have shown that disease-causing CAG repeats can form unusual DNA structures, including stable hairpins. The stability of these structures correlates with the propensity for CAG repeat expansion (Gacy et al., 1995). CAG:GAC base pairing in the stem of a hairpin contains a middle A:A base pair mismatch, which *in silico* modelling predicts will adopt an unusual Z DNA structure, perhaps via the flipping out of bases (Khan et al., 2015). This could predispose hairpin structures to both increased DNA damage and MMR protein binding. Indeed, bases damaged by ROS (e.g. 8-oxo-dG) can affect the formation and stability of hairpins, as well as having consequences on DNA repair fidelity (Volle et al., 2012). MSH2, part of the MutS mismatch recognition complexes (see Glossary, Box 1), binds directly to slipped-strand DNA structures formed by (CAG)_*n*_ *in vitro* (Pearson et al., 1997). The processing of these artificial DNA substrates by human cell extracts requires various repair factors, principally MMR proteins (including MSH2, MSH3, PMS2), although the repair outcomes depend on the starting DNA structure and not just its sequence (Panigrahi et al., 2005, 2010). It is hard to draw physiological mechanistic conclusions from these cell-free systems, but putative pathways can be identified; for example, MutSβ (MSH2/MSH3 complex) is required for repeat expansion in some assays (Nakatani et al., 2015; Stevens et al., 2013), and long-patch BER deficiency has been implicated elsewhere (Goula et al., 2009).

Further insight into CAG repeat stability has come from bacteria and yeast. These microorganisms are attractive models as they are genetically tractable, most of their DNA repair factors have been identified, and high-throughput screening assays exist for them (Bichara et al., 2006; Dixon et al., 2004; Kim et al., 2016). Repeats are unstable in these microorganisms, and seem to have a similar length threshold to that observed in human diseases, although they exhibit a propensity for repeat contractions over expansions. There is also a requirement for MMR in (CAG)_*n*_ instability: when MMR factors are knocked out, repeats are stabilised (Jaworski et al., 1995; Williams and Surtees, 2015). Given the phylogenetic conservation of MMR and other DNA repair factors from bacteria to humans (Fishel, 2015), these results might be relevant to human disease. However, bacterial and yeast cells divide in culture, and have much simpler DNA-damage response systems than human cells. They also contain eukaryotic triplet repeats out of genomic context, either on plasmids or integrated into DNA that lacks human chromatin structure and organisation. More disease-relevant data have come from multicellular organisms, as detailed below.

### Germline and somatic instability of CAG repeats

Heritable, stochastic CAG repeat expansions in germline cells (i.e. sperm or egg) are reported in many CAG and non-CAG repeat disorders (Budworth and McMurray, 2013; Durr, 2010). Given that repeat length correlates inversely with age at disease onset, this intergenerational propensity for CAG expansion explains the phenotypic observation of anticipation, whereby disease onset tends to get earlier over generations. This has been shown in all CAG repeat disorders, and is usually more marked through the paternal line (Durr, 2010), although other non-CAG trinucleotide repeat disorders, such as Huntington's disease-like 2 (HDL2) and myotonic dystrophy, show increased anticipation through the maternal line (Gövert and Schneider, 2013). Repeat length analysis has shown that significant CAG mosaicism is present in the sperm of male HD patients and that this correlates with repeat expansion on transmission (Telenius et al., 1995). Evidence from a transgenic mouse model of HD (R6/1) suggests that repeat expansion in sperm occurs after meiosis, as haploid spermatids are maturing into spermatozoa (Kovtun and McMurray, 2001). This implicates DNA repair rather than DNA replication in the DNA synthesis needed for repeat expansion.

Variations in CAG repeat length have also been found in somatic (i.e. nongermline) cells. Repeat length stability varies across cell types and can be associated with phenotype. For example, in transgenic and knock-in mouse models of HD, CAG repeat length tends to be increased in cells from the striatum, cortex and liver, but stable in cells from the cerebellum, blood and tail. Maximum expansion is observed in the striatum, which correlates well with the degeneration of striatal MSNs that underpins the disease (Gonitel et al., 2008; Lee et al., 2011; Møllersen et al., 2010). Large CAG repeat expansions have also been demonstrated in postmortem human brain neurons from HD patients, and increased expansion of repeats correlates with younger age at onset of symptoms (Kennedy, 2003; Shelbourne et al., 2007; Swami et al., 2009). DNA repair is implicated in affected neurons, as these are postmitotic. However, similar tissue-specific patterns of repeat expansion are seen in SCAs and, in these diseases, degeneration is observed in the cerebellar Purkinje neurons rather than the striatal MSNs (Chong et al., 1995; Hashida et al., 1997; Tanaka et al., 1996). The relatively high levels of expression of certain DNA repair factors in the cerebellum might prevent significant repeat expansion in this tissue. Genomic context is also important. For example, SCA7 CAG repeats in a transgenic mouse model were stable when present in complementary DNA (cDNA), but unstable if contained in a human genomic fragment. In addition, repeat stability did not correlate with neurodegeneration (Libby et al., 2003). Therefore, although somatic CAG repeat expansion can occur, and might correlate with neurodegeneration in HD, it remains unclear whether expansion drives pathology in individuals or results from downstream DNA repair defects in affected cells (Chong et al., 1995).

### Modifiers of CAG repeat stability in the genome

Various *cis* and *trans* factors can affect the stability of CAG repeats in the genome. *Cis* factors include the length of the CAG repeat, the presence of repeat interruptions, haplotype and genomic context. Longer repeats, particularly those that are over the disease-causing threshold, are more unstable and tend to expand (Fig. 3), although repeat interruptions can temper these effects (Usdin et al., 2015). Interruptions have been identified and associated with increased repeat stability, and later age at disease onset, in various SCAs and in HD (Table 1), as well as in other trinucleotide repeat disorders such as fragile X syndrome, Friedreich's ataxia and myotonic dystrophy (Chong et al., 1997; Chung et al., 1993; Eichler et al., 1994; Gao et al., 2008; Yu et al., 2011). Within the CAG repeat diseases, interruptions of a pure CAG repeat almost always involve alteration of the third base in the codon (the most tolerant of change). Interruptions were first observed in SCA1 with variable numbers of CAT codons replacing CAG (Chung et al., 1993). These reduce the stability of hairpin structures formed by pure (CAG)_*n*_ repeats in DNA, inhibiting repeat expansion, as well as inserting a number of histidines into the polyglutamine stretch of protein (Menon et al., 2013; Pearson et al., 1998; Sobczak and Krzyzosiak, 2004). More commonly, CAG repeat tracts can be interrupted by CAA codons, which alter the DNA/RNA sequence but do not affect the translated polyglutamine. In SCA2, pure CAG repeats of intermediate length (∼27-35 repeats) can expand into the disease range but repeats interrupted by CAA expand to a lesser extent and are associated with different neurological phenotypes (amyotrophic lateral sclerosis or parkinsonism, depending on the repeat number) (Charles et al., 2007; Elden et al., 2010; Yu et al., 2011). In SCA17, complex CAG repeat alleles of the TATA-box binding protein gene (*TBP*) that contain various CAA interruptions are associated with later disease onset (Gao et al., 2008). In HD, the penultimate codon of the CAG tract is usually CAA, followed by one more CAG before the (CCG)_*n*_ repeat encoding polyproline begins. The relevance of the penultimate interrupting CAA is uncertain, but its absence correlates with earlier onset (and with repeat expansion) in a few cases (Goldberg et al., 1995). Conversely, in a bacterial artificial chromosome mouse model of HD, a huntingtin (*HTT*) allele with 97 codons of alternating CAA/CAG is stable over 12 months in both germline and somatic cells, but the mice still develop a neurodegenerative pathology (Gray et al., 2008).

It appears that if a CAG repeat is above a threshold length and of appropriate codon structure and context, it is licensed to become unstable. *Trans* factors, such as DNA repair proteins, can then act on this DNA substrate to increase or decrease the repeat number in specific tissues. Work on *trans* factors has mostly been carried out in mouse models of HD. Transgenic and knock-in mouse models of HD, all with long tandem CAG tracts of >100 repeats, develop progressive neurological impairment, leading to reduced abilities in tests of motor, coordination and cognitive function (Brooks et al., 2012; Ferrante, 2009). The development of somatic CAG repeat expansion in the striatum of mouse HD models (for example, R6/1 transgenic mice carrying exon 1 of the human *HTT* gene) (Mangiarini et al., 1997) correlates with symptom development. Crossing mouse models of HD with mice that carry different DNA repair gene mutations has shown that deficiencies in specific MMR genes (e.g. *Msh2*, *Msh3*, *Mlh1* or *Mlh3*), or BER genes (e.g. *Ogg1* or *Neil1*) can abrogate somatic and/or germline CAG repeat expansion and, in some cases, ameliorate HD-like phenotypes (Budworth et al., 2015; Kovalenko et al., 2012; Pinto et al., 2013; Usdin et al., 2015; Wheeler et al., 2003). These effects seem gene specific, as knockouts of other DNA repair factors in the same pathways [e.g. of *Msh6* (MMR) or *Mpg* glycosylase (BER)] have no effect on repeat stability. In addition, results from different diseases and models have been inconsistent. For example, NER factors have been implicated in CAG repeat diseases. A study of 137 SCA3 parent-child repeat transmissions identified variants in NER factors Cockayne syndrome protein B (CSB; see Glossary, Box 1; Fig. 2C), RPA proteins and CDK7, as being associated with intergenerational repeat expansions (Martins et al., 2014). However, knockouts of different NER factors in different CAG repeat disease models have variable effects: *Csb* (*Ercc6*) knockout in HD mice promotes germline repeat instability (Kovtun et al., 2011); *XPG* (*mus201*) knockout in a *Drosophila* model of SCA3 abolishes repeat instability (Jung and Bonini, 2007); *Xpa* knockout in a SCA1 mouse reduces somatic repeat instability in many areas of the brain (although not in the cerebellum) (Hubert et al., 2011). The reasons for these differences are not fully understood, although the human homologues might not contain relevant variation, and some repair factors have additional functions outside NER; for example, CSB has chromatin remodelling and transcriptional regulation activities.

### Downstream deficits in DNA repair in CAG repeat diseases

**Fig. 4.**
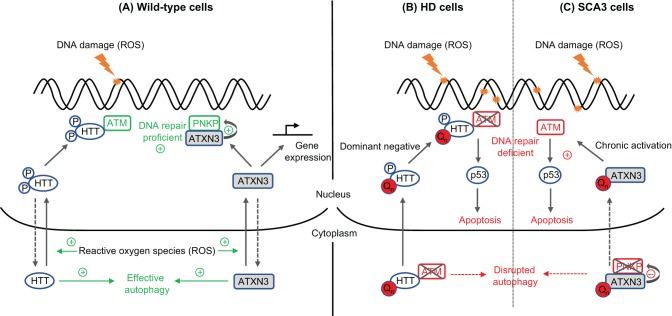
**Putative roles of HTT and ATXN3 in DNA repair and how HTT and ATXN3 polyglutamine expansions might lead to DNA damage and apoptosis.** (A) Wild-type HTT and ATXN3 proteins have various roles in the DNA damage response, as illustrated. ROS that cause DNA damage also induce the nuclear translocation of both HTT and ATXN3 (solid arrows), as well as specific HTT phosphorylation. In the nucleus, HTT is recruited to sites of DNA damage by ATM, and can act as a scaffold for DNA repair processes. Nuclear ATXN3 can bind to and stimulate the DNA end-processing repair factor, PNKP, as well as altering gene expression as part of the cellular stress response. HTT and ATXN3 also have functions in regulation of autophagy. Repair processes and their associated factors are shown in green. (B) In HD or (C) in SCA3, disease-length polyglutamine expansions (depicted as red, Q_n_) can inhibit DNA repair processes, leading to the accrual of DNA damage in cells. In both of these diseases, the mutated proteins can sequester DNA repair factors in the cytoplasm (ATM in HD, PNKP in SCA3), away from sites of DNA damage. Persistent DNA damage and signalling can result in p53-mediated apoptosis; in HD, via dominant-negative hypophosphorylated mutant HTT at sites of DNA damage; in SCA3 via chronic activation of ATM by mutant ATXN3. Nonfunctional ATM and PNKP are crossed in the figure. ATM, ataxia telangiectasia mutated; ATXN3, ataxin-3; HD, Huntington's disease; HTT, huntingtin; PNKP, polynucleotide kinase 3′-phosphatase; ROS, reactive oxygen species; SCA3, spinocerebellar ataxia type 3.

The proteins encoded by CAG repeat-containing genes have a wide range of functions in different cellular processes (Table 2). However, most are ubiquitously expressed and have links to transcriptional regulation, tying in with earlier work that identified polyglutamine stretches of 10-30 amino acids as being potent transactivators (Gerber et al., 1994). The recent realisation that repeat length polymorphism in DNA can modulate gene expression suggests that expanded repeats might affect transcription through both DNA- and protein-mediated mechanisms (Gymrek et al., 2015). The target genes that are differentially expressed as the result of repeat instability are not known. Fibroblasts, lymphoblasts or lymphocytes cultured from patients with HD or other neurodegenerative conditions accrue more DNA damage than wild-type controls; this could be caused by defective DNA repair (Moshell et al., 1980; Robison and Bradley, 1984; Scudiero et al., 1981). In support of this hypothesis, various polyglutamine-containing proteins, including HTT, androgen receptor (AR), ataxin (ATXN) 1, ATXN2 and ATXN3, have been shown to have roles in DNA repair (Table 2), and there is evidence that at least some of the pathogenesis of CAG repeat expansion might arise from the loss of wild-type protein function (Ashkenazi et al., 2017; Chatterjee et al., 2015; Gao et al., 2015; Saudou and Humbert, 2016; Zeitlin et al., 2000). As an example, regulated phosphorylation of HTT at various sites is involved in the DNA damage response. The N-terminus of HTT (particularly methionine 8) can act as a direct sensor of oxidative stress, leading to its phosphorylation at serines 13 and 16 and translocation from its usual cytoplasmic location to the nucleus (DiGiovanni et al., 2016). In the nucleus, HTT is recruited to sites of DNA damage, in a process dependent on ATM serine/threonine kinase (ATM), and might function as a scaffold for DNA repair complexes (Fig. 4A). Analysis of patient fibroblasts shows that an expanded polyglutamine in HTT does not prevent its recruitment to sites of DNA damage, but is associated with increased DNA damage, consistent with a dominant-negative effect on repair (Fig. 4B) (Maiuri et al., 2016). In addition, wild-type HTT is phosphorylated by cyclin-dependent kinase (CDK) 5 on serines 1181 and 1201 in response to DNA damage, and this seems to have a protective role in inhibition of p53 (TP53)-induced cell death. Loss of this protective phosphorylation in ageing and/or disease-associated neurons could lead to increased cell death, although the exact molecular mechanisms involved are not understood (Fig. 4B) (Anne et al., 2007).

**Table 2.**
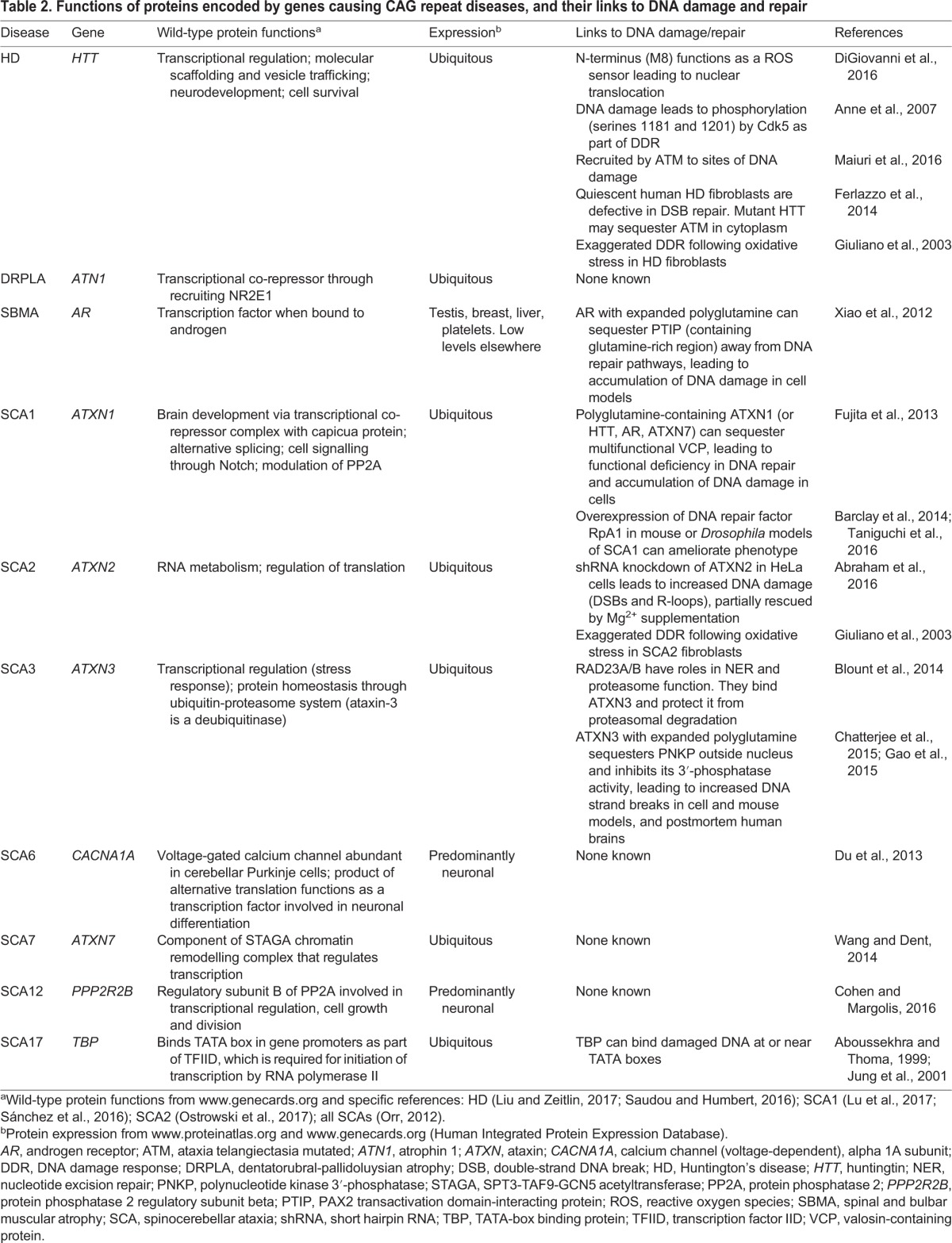
**Functions of proteins encoded by genes causing CAG repeat diseases, and their links to DNA damage and repair**

Recent work on ATXN3 has reinforced the importance of subcellular localisation and protein-protein interactions in the regulation of DNA repair. ATXN3 is a deubiquitinase that has a neuroprotective role mediated by its regulation of autophagy (via wild-type polyglutamine; see Glossary, Box 1) (Ashkenazi et al., 2017), the ubiquitin-proteasome system, and DNA repair. Heat-shock or oxidative stress can stimulate the nuclear translocation of ATXN3, where it has roles in transcriptional regulation, possibly through its deubiquitinase activity, and DNA repair (Fig. 4A) (Orr, 2012). The latter was first suggested by the interaction of ATXN3 with human RAD23 homologues in a yeast two-hybrid screen, as RAD23 proteins are involved in NER (Fig. 2C) (Wang et al., 2000). More recently, ATXN3 has been shown to protect cells from DNA damage through its interaction with polynucleotide kinase 3′-phosphatase (PNKP), a key enzyme involved in the processing and repair of DNA strand breaks (Figs 1 and 2) (Chatterjee et al., 2015). Rare mutations in PNKP can cause ataxic syndromes (Bras et al., 2015). Interestingly, the expansion of a polyglutamine tract in ATXN3 leads to both the sequestration of PNKP outside the nucleus and the inhibition of nuclear PNKP activity, which together lead to impaired DNA repair. Persistent DNA damage (and DNA damage signalling through ATM) is observed in mouse and cellular models of SCA3, as well as in postmortem human SCA3 brains, and this can trigger cell death through p53-mediated and other pathways (Fig. 4C) (Chatterjee et al., 2015; Gao et al., 2015).

A similar theme is continued in cell models of SBMA and SCA1. Expanded polyglutamine within AR or ATXN1, respectively, lead to the sequestration of proteins involved in DNA repair and the subsequent accumulation of DNA damage (Fujita et al., 2013; Xiao et al., 2012). As well as being intrinsically deleterious to cells, unrepaired DNA damage also leads to persistent activation of the DDR, which in itself can be toxic, as seen in other neurodegenerative disorders (Hoch et al., 2016). The importance of accurate and timely DNA repair in the cerebellum has long been noted, given that mutations in enzymes such as aprataxin and tyrosyl DNA phosphodiesterase 1 (TDP1), responsible for processing damaged DNA ends to permit repair (Fig. 1), lead specifically to cerebellar neurodegeneration (Ward and La Spada, 2015).

Therefore, there is increasing evidence in a range of CAG repeat diseases that defective DNA repair might be involved in disease pathogenesis. An expanded polyglutamine tract could lead to the inactivation and/or inappropriate sequestration of repair proteins, such that DNA damage builds up in neurons. If the wild-type proteins themselves have functions in DNA repair, then pathogenesis could be the result of a combination of loss of function and dominant-negative gain of function (Fig. 4). Accrual of DNA damage would also predispose CAG repeats, which are already susceptible to damage as discussed above, to further strand breaks, the repair of which could result in further repeat expansion, thus setting up a toxic cycle (Fig. 3C,D).

## Conclusion

CAG repeat disorders consist of a set of overlapping diseases that are linked by pathogenic repeat expansion, neurodegeneration and lack of disease-modifying therapies. Although some of the causative mutations have been known for 25 years, very little progress has been made in translating findings from cell and animal models of these diseases into new treatments. The recent discovery of disease-modifying genetic variants in HD and SCAs has shown the power of ‘natural’ clinical trials, which capture information on modifying variants that have been ‘crossed’ onto disease-causing CAG repeat mutations present in the population (Holmans et al., 2017). Excitingly, many of the identified disease-modifying variants converge on specific DNA repair pathways, such as MMR. Understanding why this is the case could illuminate the link between CAG repeat expansion and neurodegeneration. Variants could be influencing CAG repeat pathogenesis by affecting the CAG repeat itself and/or by modulating the downstream DNA damage that results from a defective polyglutamine-containing protein. Ongoing GWAS and the future whole-exome or genome sequencing of individuals with CAG repeat diseases promise to yield more leads, which will need to be validated in human cell models to gain a greater understanding of the underlying molecular mechanisms involved.

Common variation in DDR pathways has also been associated, via GWAS, with a range of other diseases. In the neuropsychiatric field, variation in the MMR gene *MLH1* has been associated with autism, schizophrenia and lithium-responsive bipolar disorder (Autism Spectrum Disorders Working Group of The Psychiatric Genomics Consortium, 2017; Ripke et al., 2014; Song et al., 2016), while the broader DDR has been implicated in frontotemporal dementia (Ferrari et al., 2017). Beyond neuropsychiatry, DDR genes have also been associated with lipid metabolism (*MSH3* and *FAN1* loci) (Weissglas-Volkov et al., 2013), reproductive ageing (Day et al., 2015) and longevity (Shadyab and LaCroix, 2015). Inherited mutations in multiple DDR genes are associated with familial cancers, but there is scant evidence for common DDR variation increasing cancer risk in nonfamilial disease. Possible contributions of the DDR genes to testicular cancer (Litchfield et al., 2015) and in a grouped analysis of lung, ovary, prostate, breast and colorectal cancers (Scarbrough et al., 2016) have been identified, but the lack of DDR gene and pathway associations in the many very large cancer GWAS implies that variants in these pathways are, at most, likely to have small effect sizes in these diseases. Therefore, variation in DDR pathways is not specific to CAG repeat disorders and could impact multiple diseases in different ways.

The development of olaparib, a poly(ADP-ribose) polymerase (PARP; see Glossary, Box 1; Fig. 2) inhibitor, as a useful antineoplastic drug has shown that therapeutic manipulation of the DNA damage response is feasible in humans (Brown et al., 2017; Pearl et al., 2015). Cross-pollinating advances made in the cancer field, such as the development of synthetic lethality screens (see Glossary, Box 1) in the DDR, with novel genetic leads identified from studying CAG repeat diseases, could help develop drugs more rapidly. Furthermore, therapeutic leads based on genetic discoveries linked directly to human disease phenotypes are more likely to be translated into effective disease-modifying clinical treatments (Nelson et al., 2015; Plenge et al., 2013). Finally, the build-up of DNA damage in ageing neurons, potentially exacerbated by defective DNA repair processes, could represent a broader paradigm for neurodegenerative pathogenesis, making the findings of CAG repeat disease research more widely applicable.
